# Supplementary material for: Robustness of the honeybee neuro-muscular octopaminergic system in the face of cold stress
Source: Front Physiol. 2022 Sep 27;13:1002740. doi: 10.3389/fphys.2022.1002740 (PMC9551396; doi:10.3389/fphys.2022.1002740)

Robustness of the honeybee neuro-muscular octopaminergic system in the face of cold stress - Supplementary Material

1 SUPPLEMENTARY TABLES AND FIGURES

1.1 Tables

Results of the statistical analysis (Mann-Whitney U test) of dopamine (DA) and serotonin (5-HT) quantification in workerbee flight muscles (DV+DL) and mesometathoracic ganglion (MMTG) under cold stress. Serotonin was only detectable in the MMTG but not the flight muscles. Tyramine was not quantifiable in many samples and was therefore not considered. For visualization see Figure S1.

bees	tissue	time	amine	$N_{34\,°C}$	$N_{10\,°C}$	U	p	z	r
one week old	DV+DL	30 min	DA	8	10	26.00	0.24	-1.18	-0.28
one week old	DV+DL	120 min	DA	10	9	45.00	1.00	0.00	0.00
one week old	MMTG	30 min	DA	9	9	50.00	0.44	-0.78	-0.18
one week old	MMTG	30 min	5-HT	9	9	55.00	0.22	-1.22	-0.29
one week old	MMTG	120 min	DA	9	9	38.00	0.86	-0.17	-0.04
one week old	MMTG	120 min	5-HT	7	9	47.00	0.11	-1.58	-0.40
forager	DV+DL	30 min	DA	10	10	37.00	0.35	-0.93	-0.21
forager	DV+DL	120 min	DA	10	10	48.00	0.91	-0.11	-0.02
forager	MMTG	30 min	DA	10	10	48.00	0.91	-0.11	-0.02
forager	MMTG	30 min	5-HT	10	10	36.00	0.32	-1.00	-0.22
forager	MMTG	120 min	DA	10	10	76.00	0.05	-1.94	-0.43
forager	MMTG	120 min	5-HT	8	6	31.00	0.41	-0.82	-0.22

1.2 Figures

Dopamine (DA) and Serotonin (5-HT) concentrations in the flight muscles (A, C) and the MMTG (B, D) under cold stress conditions. For statistics see Table S1. For each group/data-set median ± IQR (left part) and individual data points (right part) are shown.

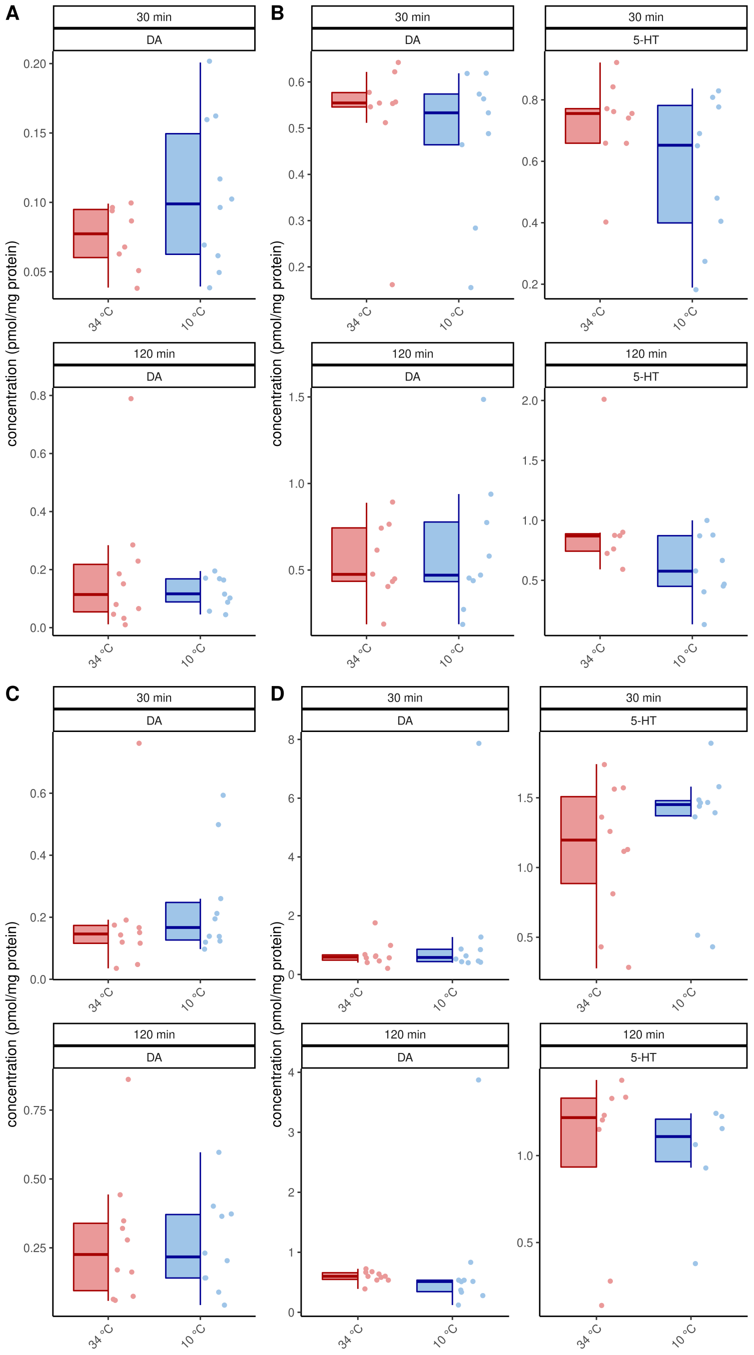

Supplement: Supplementary file 1 [file DataSheet1.PDF]
